# Preimplantation exposure to viral dsRNA impairs decidualization leading to altered placental development and fetal growth restriction

**DOI:** 10.1530/RAF-26-0018

**Published:** 2026-08-03

**Authors:** Jacy N Scott, Thomas M Rawlings, Laiba Jamshed, Maria Tryfonos, Zhonghua Tang, Rolando Garcia-Milian, Eric Y Huang, Paul J Brighton, Jan J Brosens, Emma S Lucas, Mancy Tong

**Affiliations:** ^1^ Department of Obstetrics, Gynecology and Reproductive Sciences, Yale School of Medicine, New Haven, Connecticut, USA; ^2^ Division of Biomedical Sciences, Warwick Medical School, University of Warwick, Coventry, United Kingdom; ^3^ Harvey Cushing/John Hay Whitney Medical Library, Yale School of Medicine, New Haven, Connecticut, USA; ^4^ School of Medicine and Population Health, University of Sheffield, Sheffield, UK

**Keywords:** infection, inflammation, endometrium, decidua, trophoblast, placentation, resorption, SGA, Toll-like receptor

## Abstract

**Graphical Abstract:**

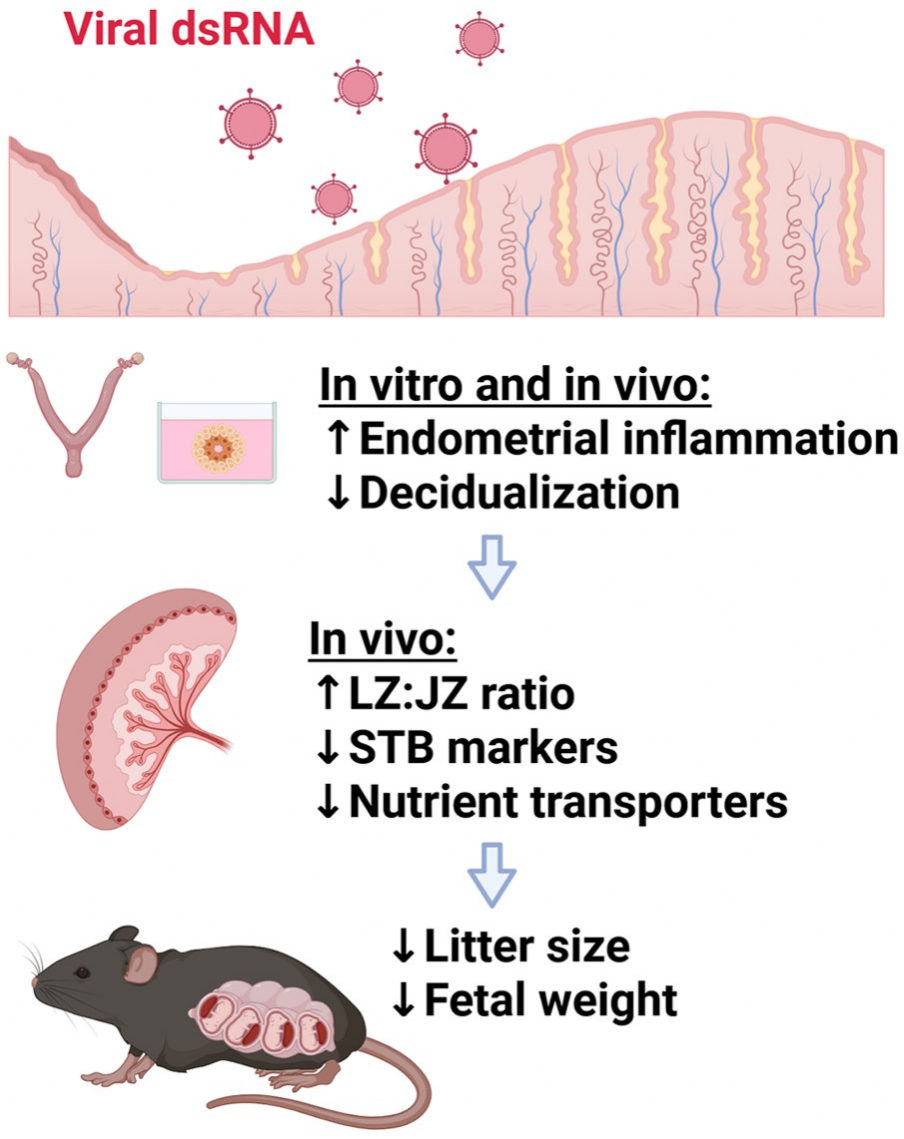

**Abstract:**

Viral infections are associated with early pregnancy loss and later gestational complications, including preeclampsia and fetal growth restriction. While viral components, including viral double-stranded RNA (dsRNA), have been shown to affect placental trophoblast function, how these components may affect maternal endometrial function is less understood. Endometrial stromal cells make up the bulk of the human endometrium, and they undergo a specialized hormone-driven differentiation process termed decidualization, monthly in preparation for pregnancy. We have previously shown that these stromal cells exhibit robust inflammatory responses to bacterial LPS and viral dsRNA. The goals of this study are to determine the effects of prior viral dsRNA exposure on endometrial decidualization and whether this affects subsequent placentation and pregnancy outcomes. Using a combination of 2D endometrial stromal cell cultures, 3D human endometrial assembloids, and mouse models of decidualization and pregnancy, we demonstrated that exposure to viral dsRNA (Poly(I:C)) 48 h prior to hormonal stimulation impaired decidualization *in vitro* and *in vivo*. Similarly, preimplantation exposure to Poly(I:C) on GD0.5 in timed-mated dams inhibited decidualization, reduced implantation success, and induced fetal growth restriction. This was associated with altered placentation, in particular, formation of the Syn-TII cells in the labyrinth zone, and dysregulated expression of glucose and amino acid transporters in the placenta. This work emphasizes the importance of preconception endometrial health for successful pregnancy and identifies a novel mechanism by which prior viral infection can lead to implantation failure and later pregnancy complications.

**Lay summary:**

Viral infections increase the risk of early pregnancy loss and later complications, such as poor fetal growth. While viruses are known to affect the placenta, less is understood about their impact on the uterine lining, which must be healthy for pregnancy to begin. The uterine lining is made up of cells that undergo changes each month called decidualization, which is driven by hormones to help with implantation. In this study, we examined how early exposure to viral infection affects decidualization and pregnancy outcomes. Using cell cultures, 3D uterine models, and mouse studies, we found that exposure to viral factors impaired decidualization, reduced embryo implantation, and disrupted fetal growth. These effects were linked to abnormal placental development and altered nutrient transport to the fetus. Overall, our findings show that uterine health before conception is critical for successful pregnancy and identify a mechanism by which early maternal viral infections may lead to pregnancy complications. This provides exciting opportunities to screen and prevent complications much earlier on in pregnancy.

## Introduction

Maternal infections with a range of DNA and RNA viruses, including cytomegalovirus, HIV, and influenza, are associated with an increased risk of implantation failure and miscarriage (Bloom-Feshbach *et al.* 2011, Kim *et al.* 2012, Ezechi *et al.* 2013, Giakoumelou *et al.* 2016). Meanwhile, the roles of other common viruses, such as human simplex viruses (HSV-1 and HSV-2), human papillomavirus, and hepatitis B, in miscarriage remain controversial (Giakoumelou *et al.* 2016). Existing maternal viral infections have also been associated with an increased risk of fetal growth restriction (Iqbal *et al.* 2010, Longo *et al.* 2014) and preeclampsia by 2–3 times (Gaccioli *et al.* 2020, Conde-Agudelo & Romero 2022, Motomura *et al.* 2024). Viruses can gain access to the uterus by ascending from the lower reproductive tract or hematogenous transmission where they can cause endometritis and induce inflammation at the maternal–fetal interface (deciduitis and villitis) (Racicot & Mor 2017). Fetal placental trophoblasts are known to express Toll-like receptors, a conserved family of innate immune receptors that recognize bacterial and viral components, including single-stranded and double-stranded RNA (dsRNA), and their inflammatory responses are well reported (Abrahams *et al.* 2004, Chatterjee *et al.* 2012, Pudney *et al.* 2016, Motomura *et al.* 2024). In comparison, how the maternal endometrium, or decidua, during pregnancy, responds to viral infections is much less understood.

The endometrium is a highly dynamic tissue that plays a central role in dictating uterine receptivity for blastocyst implantation. In addition to being a cause for miscarriage (Brosens *et al.* 2022, Muter *et al.* 2025), recent studies have also implicated the endometrium/decidua in the pathogenesis of later obstetric complications, including preeclampsia and fetal growth restriction (Conrad *et al.* 2017, James-Allan *et al.* 2018, Dunk *et al.* 2019, Garrido-Gomez *et al.* 2022). Endometrial stromal cells form the majority of the cells within the human endometrium and they undergo major proliferation and differentiation monthly in response to changes in circulating levels of estrogen and progesterone. In the luteal phase, increased estrogen and progesterone stimulates endometrial stromal cells to undergo decidualization, a specialized differentiation process that induces transcriptional reprogramming of these cells to form highly secretory, stress-resistant decidual cells. These epithelioid cells secrete many immunomodulatory mediators and growth factors that enable them to play a central role in coordinating nearby cell types to support implantation. This includes interacting with overlying epithelial cells to stimulate endometrial receptivity; regulating recruitment and activity of natural killer cells and other immune cells; and stimulating endometrial vasculogenesis (Gellersen & Brosens 2014, Muter *et al.* 2021). Indeed, it has been reported that the endometrium can act as a biosensor of embryo quality: rejecting and aborting low-quality blastocysts to enable the uterus to conserve resources for viable pregnancies (Salker *et al.* 2010, Teklenburg *et al.* 2010, Kong *et al.* 2021, Muter *et al.* 2025).

Decidual transformation, while initiated cyclically in humans and other menstruating mammals independent of fertilization, only completes fully upon successful blastocyst implantation. Throughout pregnancy, decidualized stromal cells continue to play a central role in modulating trophoblast invasion and placental function to ensure pregnancy success (Menkhorst *et al.* 2012, James-Allan *et al.* 2018). Moreover, to protect the fetus from external infections while simultaneously tolerating the semi-allogeneic placenta, the decidua harbors a specific milieu of innate and adaptive immune cells whose composition changes dynamically as gestation progresses (Canavan & Simhan 2007, Nancy *et al.* 2012, Mori *et al.* 2016, Crespo *et al.* 2020). In the presence of bacterial/viral infection, decidual immune cell recruitment and functions change, leading to heightened inflammation and potentially placental dysfunction and/or fetal demise (Canavan & Simhan 2007, Castro-Leyva *et al.* 2017, Crespo *et al.* 2020). Indeed, a previous study reported that systemic inflammation induced by viral dsRNA on gestational day 13 onwards leads to endothelial cell dysfunction and hypertension in mice (Chatterjee *et al.* 2012), although this is likely mediated by a direct inflammatory effect on the placenta rather than decidual dysfunction.

We previously demonstrated that human endometrial stromal cells mount a robust inflammatory response to bacterial lipopolysaccharide and viral dsRNA (Tong *et al.* 2023). Viral dsRNA induces activation of NFKB leading to the secretion of many proinflammatory cytokines and chemokines including TNF-α and IL-8 that have been associated with deleterious pregnancy outcomes (Cotechini *et al.* 2014). Interestingly, we observed that decidualization increased the expression of IKB, leading to inhibition of Toll-like receptor signaling and reduced inflammatory responses by human endometrial stromal cells (Tong *et al.* 2023). Dampened release of chemotactic signals following decidualization has also been reported by others (Nancy *et al.* 2012).

The objective of this study is to determine the downstream consequences of early viral dsRNA exposure by endometrial stromal cells. We used a combination of 2D and 3D human endometrial stromal cell cultures, as well as mouse models of decidualization and pregnancy, to test the hypothesis that viral dsRNA-induced inflammation perturbs decidualization, leading to a precarious fetal–maternal interface prone to miscarriage and unable to properly support subsequent placental and fetal development.

## Methods

### Human study approvals

Anonymized endometrial biopsies were obtained from women attending the Implantation Research Clinic, University Hospitals Coventry and Warwickshire National Health Service (UHCW NHS) Trust, using a Wallach Endocell™ endometrial sampler. Written informed consent was obtained in compliance with the Declaration of Helsinki (World Medical Association 2013). The study was approved by the Arden Tissue Bank at UHCW NHS Trust (NHS Research Ethics Committee approval: 18/WA/0356). Samples were obtained during the luteal phase of ovulatory menstrual cycles and timed relative to the pre-ovulatory LH surge (LH + 5 to LH + 9), as determined by commercially available ovulation test kits.

### 2D culture of endometrial stromal cells

A well-characterized telomerase-immortalized human endometrial stromal cell line (ATCC: CRL-4003) was used for validation of inflammatory and decidualization responses (Krikun *et al.* 2004). Stromal cells were cultured in phenol red-free DMEM/F12 medium (Sigma-Aldrich, USA; D2906) supplemented with 10% fetal bovine serum (FBS; R&D Systems, USA, S11550), 1% HEPES, and 1% penicillin/streptomycin at 37°C in humidified 95% air/5% CO_2_. Cells were passaged 1:3 when 90% confluent and cells used for this study were under ten passages.

Endometrial stromal cells were pretreated with 1 μg/mL Poly(I:C) in phenol red-free DMEM/F12 supplemented with 2% charcoal-stripped FBS (Thermo Fisher, USA, A33821-01) for 48 h before the addition of decidualizing media (Dec: 10 nM 17β-estradiol (Cayman Chemicals, USA, 10006315), 1 μM MPA (Cayman Chemicals, 23664), and 0.5 mM 8-bromo-cAMP (Enzo Life Sciences, USA, BML-CN115-0050)) for up to 96 h with the change in media every 48 h (Krikun *et al.* 2004, Tong *et al.* 2022, Tong *et al.* 2023). Poly(I:C) was supplemented throughout media changes.

### 3D culture of endometrial assembloids

Endometrial biopsies were finely minced and digested enzymatically with 500 μg/mL collagenase type Ia (Merck Life Science, Germany, C9891) and 100 μg/mL DNase I (Lorne Laboratories LTD, UK, LS002060) for 1 h. After filtering through a 40 μm cell sieve, stromal cells were cultured in growth medium (DMEM/F12 supplemented with 10% dextran-coated charcoal-stripped FBS, 2 mM L-glutamine, 1 nM β-estradiol (Merck Life Science, E2758), 2 μg/mL recombinant human insulin (Merck Life Science, 91077C), and antibiotic-antimycotic). Growth factor-reduced Cultrex RGF Basement Membrane Extract, type 2 (Biotechne, USA), was added to the freshly isolated endometrial gland fragments at a ratio of 1:20 (cell pellet: Cultrex). About 20 μL droplets were cured and overlaid with expansion medium (ExM: Advanced DMEM/F12 supplemented with N2, B27, 2 mM L-glutamine, 500 nM A83-01, 500 ng/mL R-respondin-1, 10 mM nicotinamide, 1.25 mM N-acetyl-L-cysteine, 100 ng/mL Noggin, 100 ng/mL FGF-10, 50 ng/mL HGF, 50 ng/mL EGF, and antibiotic-antimycotic) (Turco *et al.* 2017, Rawlings *et al.* 2021).

Twenty-four hours after stromal cells and gland organoids were established, assembloids were established as described previously (Rawlings *et al.* 2021). Stromal cells and gland organoids were digested into a single cell suspension and mixed at a ratio of 1:1 (v/v) before adding ice-cold PureCol EZ Gel (Sigma-Aldrich) at a ratio of 1:20 (cell pellet: hydrogel). The suspension was aliquoted in 20 μL droplets. Following polymerization, droplets were overlaid with ExM with 10 nM β-estradiol. Assembloids were grown in ExM with E2 for 4 days, followed by a 4-day decidual time course. To induce decidualization, ExM was replaced with minimal decidualization medium (MDM) containing 1 nM β-estradiol, 1 μM medroxyprogesterone acetate (MPA), and 0.5 mM 8-bromo-cAMP. For Poly(I:C) treatment, assembloids were pretreated with 1 μg/mL Poly(I:C) (High MW, VacciGrade^TM^, vac-pic, InvivoGen) after 2 days of ExM with E2 (Day 2) and continued upon decidual induction. Poly(I:C) treatment was continued in MDM for 4 days. For consistency, assembloids grown in ExM are hereafter referred to as NT, and assembloids grown in MDM with and without Poly(I:C) are Dec and Dec + P, respectively.

### Cytokine secretion

Cell-free conditioned media from treated endometrial stromal cells and assembloids were stored at −80°C until further analysis. The following cytokines/chemokines were quantified using the Bio-Plex Pro Human Cytokine 27-plex Assay (M500KCAF0Y, Bio-Rad Laboratories, USA): FGF basic, eotaxin, G-CSF/CSF3, GM-CSF/CSF2, IFN-γ, IL-1β, IL-1ra, IL-2, IL-4, IL-5, IL-6, IL-7, IL-8, IL-9, IL-10, IL-12(p70), IL-13, IL-15, IL-17A, IP-10/CXCL-10, MCP-1/CCL2, MIP-1α/CCL3, MIP-1β/CCL4, PDGF-BB, RANTES/CCL5, TNF-α, and VEGF. Data were collected on the Luminex 200 analyzer (Yale Flow Cytometry Facility) and processed using RStudio, version 2025.05.1, Build 513 (Posit Software, USA, PBC). Cytokines with more than 50% N/A values across all samples were filtered out, and remaining proteins were log_2_-transformed. Missing values were imputed by k-nearest neighbor (k-NN) method (Beretta & Santaniello 2016). Differential expression analysis was performed by multi-group comparison ANOVA with *P* < 0.05. Qlucore Omics Explorer version 3.10.21 (Qlucore, Sweden) was used for heat maps and data visualization.

Duoset solid-phase sandwich enzyme-linked immunosorbent assay (ELISA) kits (R&D Systems) were used for the quantification of IGFBP-1 (DY871), IL-8 (DY208), MCP-1 (DY279), prolactin (DY682), and ST2 (DY523B) secretion. Assays were performed according to manufacturer’s instructions, and absorbance was measured at 450 nm. Protein concentration was obtained using a four-parameter logistic regression analysis and interpolation from the standard curve.

### RNA sequencing

Assembloid cultures were washed with PBS before digestion with 500 μg/mL collagenase I for 10 min at 37°C with regular manual shaking. Samples were washed twice in PBS and cell pellets were snap-frozen at −80°C. RNA extraction was performed using the RNeasy Micro Kit (Qiagen Manchester, UK) according to the manufacturer’s instructions. RNA concentration and purity were determined using a NanoDrop ND-1000 and all RNA samples were stored at −80°C until use.

After RNA from all assembloids had been extracted, quality control was performed on the Agilent 2100 Bioanalyzer using Total RNA Nano chips (Agilent Technologies, UK), and libraries were prepared using TruSeq RNA library preparation V2 kits (Illumina, UK). Samples were sequenced on a NextSeq 500 (Illumina), using a 75-cycle high-output cartridge and 42 bp paired-end reads. Sequence reads were aligned to Hg38 using STAR 2.5 and the number of reads in features was calculated using htseq-count (HTseq v2.0.0). Transcripts per million were calculated and used for differential gene expression analysis. Data have been deposited into NCBI GEO Omnibus (GSE335470).

### Bioinformatic analysis

Differential expression analysis was performed in R (v4.5.1) using DESeq2 (Bioconductor; v1.48.1). Read counts were normalized with the median-of-ratios method, and outlier detection and dispersion estimation were performed with default DESeq2 functions. Genes were considered differentially expressed when they satisfied both thresholds: FDR-adjusted *P* < 0.05 (Benjamini–Hochberg correction) and |log_2_ fold-change|>1.

Principal component analysis (PCA) was conducted on rlog-transformed expression values and plotted using plotPCA (DESeq2). Heatmaps were produced with pheatmap (v1.0.13) from z-score-scaled expression values. Differential gene expression was assessed for Dec vs NT and Dec + P vs Dec. Volcano plots were generated in ggplot2 (v3.5.2), and the 20 most significant genes by adjusted *P*-value were annotated using ggrepel (v0.9.6). The top 100 most variable genes were used for unsupervised clustering. Gene set enrichment analysis (GSEA) was performed using MSigDB Hallmark pathways on unfiltered differentially expressed genes. Genes were ranked by signed −log10 adjusted *P*-value, and enrichment significance was assessed using a permutation-based approach with Benjamini–Hochberg correction.

### Animal study approval

All animal studies were conducted in accordance with the National Institutes of Health guide for the care and use of laboratory animals and approved by Yale University’s Institutional Animal Care and Use Committee (Protocol #2023-20492). Mice were housed in standard cages at a pathogen-free animal facility following a 12 h light:12 h darkness cycle and provided food and water *ad libitum*.

### Mouse decidualization model

Female C57/BL6J mice (Jackson Laboratories) were ovariectomized at 4–5 weeks, and after resting for 10 days to deplete endogenous sex hormones, decidualization was induced as previously described (Whirledge *et al.* 2015, Tong *et al.* 2022). Briefly, mice were administered 100 ng estradiol (E2, Sigma-Aldrich, E2758) subcutaneously (sc) for three days. After 2 days of rest, mice were administered 6.7 ng E2 and 1 mg progesterone (P4, P8783) sc for a total of 5 consecutive days. On day 3 of combined E2 and P4 administration, 400 μg Poly(I:C) (20 mg/kg) or an equivalent volume of saline was injected intraperitoneally and 3 h later, mice were anesthetized and a deciduogenic stimulus of 25 μL sesame oil was injected into the right uterine horn. After recovery, mice continued to receive sc injections of E2 and P4 daily for 2 days. Forty-eight hours after induction of decidualization, mice were euthanized and the two uterine horns were dissected separately and snap-frozen for downstream analysis (Fig. 3A).

### Timed matings

Female C57BL/6 mice (6–12 weeks) were housed with C57BL/6 studs in a 2:1 ratio and on the morning a copulatory plug is observed (0.5 dpc), 400 μg Poly(I:C) (20 mg/kg) or an equivalent volume of saline was injected intraperitoneally. On gestational day (GD) 5.5, uteri were dissected, weighed, and the number of pups and resorptions counted. From GD15.5 uteri, the decidua, placenta, and fetuses were separated, weighed, and snap frozen or fixed in 4% paraformaldehyde overnight for downstream analysis. Wet weights of fetuses were recorded before collecting their tails for sex determination.

### Quantitative RT-PCR

Total RNA was extracted using TRIzol^TM^ Reagent (Thermo Fisher) according to the manufacturer’s instructions. Total RNA concentration and purity were assessed using the NanoDrop One Spectrophotometer (Thermo Fisher). cDNA synthesis was performed following manufacturer’s guidelines using the SuperScript II kit (Invitrogen, USA) with removal of DNA contamination using DNase I. qPCR was performed using Luna® Universal qPCR Master Mix (NEB) with 5–20 ng of cDNA as the template, following the manufacturer’s instructions. *Rplp0* and *Tuba* were used as housekeeper genes in mice uteri and placenta, respectively. All primer sequences used in this study are listed in Table 1. Relative gene expressions were calculated using the 2^–ΔΔCt^ method.

**Table 1:** Primers used in this study.

Target gene	Sequence
qPCR	
*Lat1*/*Slc7a5*	5^′^-CTGCTGACACCTGTGCCATC-3^′^ 5^′^-GGCTTCTTGAATCGGAGCC-3^′^
*Lat2*/*Slc7a8*	5^′^-CCAGTGTGTTGGCCATGATC-3^′^ 5^′^-TGCAACCGTTACCCCATAGAA-3^′^
*Glut1*/*Slc2a1*	5^′^-GCTTATGGGCTTCTCCAAACT-3^′^ 5^′^-GGTGACACCTCTCCCACATAC-3^′^
*Glut4*/*Slc2a4*	5^′^-GTAACTTCATTGTCGGCATGG-3^′^ 5^′^-AGCTGAGATCTGGTCAAACG-3^′^
*Mct4*	5^′^-GGCTGGCGGTAACAGAGTA-3^′^ 5^′^-CGGCCTCGGACCTGAGTATT-3^′^
*Synb*	5^′^-TCCGGAAAGGGACCTGCCCA-3^′^ 5^′^-CAGCAGTAGTGCGGGGTGCC-3^′^
*Ctsq*	5^′^-AATTGGCTATGGTTATGTGGGA-3^′^ 5^′^-TCACACAGTAGGGTATTGGG-3^′^
*Tpbpa*	5^′^-GCCAGTTGTTGATGACCCTGA-3^′^ 5^′^-CCCATCGCCACTCTCTGTGT-3^′^
*Pl1*/*Prl3d1*	5^′^-CTCACTTGGAGCCTACATTG-3^′^ 5^′^-ACCAGGTGTTTCAGAGGTTC-3^′^
*Tnfa*	5^′^-CTATGTCTCAGCCTCTTCTC-3^′^ 5^′^-CCATTTGGGAACTTCTCATC-3^′^
*Bmp2*	5^′^- TTG CAC ACT TGC TGT CTG TT-3^′^ 5^′^- GTT CTC ACG GAT TGG ACA AC -3^′^
*Dprp*	5^′^- TTA TGG GTG CAT GGA TCA CTC C-3^′^ 5^′^- CCC ACG TAA GGT CAT CAT GGA T -3^′^
*Wnt4*	5^′^- ATA CGC CAT CTC TTC AGC AG-3^′^ 5^′^- GCC TCG TTG TTG TGA AGA TT-3^′^
*Tuba*	5^′^-TGTCCTGGACAGGATTCGC-3^′^ 5^′^-CTCCATCAGCAGGGAGGTG-3^′^
*Rplp0*	5^′^-GGACCCGAGAAGACCTCCTT-3^′^ 5^′^-GCACATCACTCAGAATTTCAATGG-3^′^
PCR	
*Sx*	5^′^-GATGATTTGAGTGGAAATGTGAGGTA-3^′^ 5^′^-CTTATGTTTATAGGCATGCACCATGTA-3^′^
*Kdm5c/d*	5^′^-CCACTGCCAAATTCTTTGG-3^′^ (reverse) 5^′^-TGGGGATGTCAAGATGGAAT-3^′^ (forward) 5^′^-ATATGCTCTCGTGGGGATGA-3^′^ (forward)

### Sex determination

Sex determination was performed as described from DNA (McFarlane *et al.* 2013) and cDNA (Bosso-Lefevre *et al.* 2023). Total DNA was extracted from tail biopsies using alkaline lysis reagent (25 mM NaOH, 0.2 mM disodium EDTA). PCR was performed using Hotstart Taq (New England Biolabs, USA, M0495S) and mouse *Sx* primers (Table 1). Standard PCR parameters were used: initial denaturation at 94°C for 2 min; 35 cycles with 94°C for 30 s, 57°C for 30 s, and 72°C for 30 s, followed by final elongation at 72°C for 5 min. For sex determination using cDNA, *Kdm5c/d* reverse, and *Kdm5c* and *Kdm5d* forward primers were used together with Luna® Universal qPCR Master Mix (NEB). The thermal cycling program was as follows: initial denaturation at 95°C for 1 min; 35 cycles of 95°C for 15 s, 54°C for 30 s, and 68°C for 1 min, followed by a final extension at 68°C for 5 min. PCR products were visualized following electrophoresis with a 100 bp DNA ladder (NEB) on 2% agarose gels stained with SYBR™ Safe DNA Gel Stain (Thermo Fisher).

### Statistical analysis

The number of independent experiments that data were pooled from is indicated in the figure legends as ‘*n* = ’. Three sets of patient-derived assembloids were generated and treated for RNA sequencing. All other experiments were performed at least five times. All data are reported as mean ± SEM. Statistical significance was set at *P* < 0.05 and determined using Prism Software (GraphPad). For normally distributed data, significance was determined using either one-way analysis of variance (ANOVA) for multiple comparisons or a *t*-test. For data not normally distributed, significance was determined using a non-parametric multiple comparison test or the Wilcoxon matched-pair signed-rank test.

## Results

### Poly(I:C) exposure increased inflammation and impaired decidualization in human endometrial stromal cells

**Figure 1:**
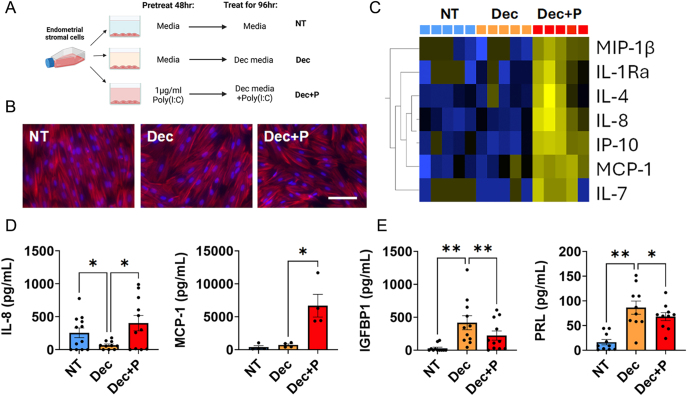
Poly(I:C) induces inflammation and inhibits decidualization in human endometrial stromal cells. (A) A telomerase-immortalized human endometrial stromal cell line was pretreated with 1 μg/mL Poly(I:C) for 48 h before the addition of a decidualization cocktail (10 nM estradiol, 1 μM medroxyprogesterone acetate, and 500 μM 8-bromo cAMP) for 96 h. (B) Phalloidin staining (red) showing morphological changes associated with decidualization (blue = nuclei, scale bar = 100 μm). (C) Heatmap of factors secreted by endometrial stromal cells under the three treatment conditions; yellow is upregulated, and blue is downregulated (*n* = 4). (D and E) ELISA quantification of endometrial secretion of IL-8, MCP-1, IGFBP-1, and prolactin (PRL, *n* = 10). *P < 0.05, **P < 0.01.

To determine the impact of viral dsRNA (Poly(I:C)) on decidualization, human endometrial stromal cells were pretreated with Poly(I:C) for 48 h prior to decidualization for 96 h (Fig. 1A and B). Inflammatory cytokine and chemokine secretion were profiled using a Bio-Plex Pro Human Cytokine 27-plex Assay. Poly(I:C) exposure significantly upregulated the secretion of MIP-1β, IL-1Ra, IL-4, IL-8, IP-10, MCP-1, and IL-7 by human endometrial stromal cells (Fig. 1C). We validated IL-8 and MCP-1 secretion by ELISA: Poly(I:C) induced secretion by 5.8 ± 1.6-fold and 12.1 ± 4.9-fold, respectively (Fig. 1D). Moreover, Poly(I:C) pretreatment significantly impaired endometrial stromal cell decidualization as evidenced by reduced secretion of two classic markers, IGFBP-1 (51.9 ± 8.9%, *P* < 0.01) and prolactin (PRL, 9.9 ± 10.7%, *P* < 0.05, Fig. 1E).

### Poly(I:C) exposure increased inflammation and impaired decidualization in endometrial assembloids

**Figure 2:**
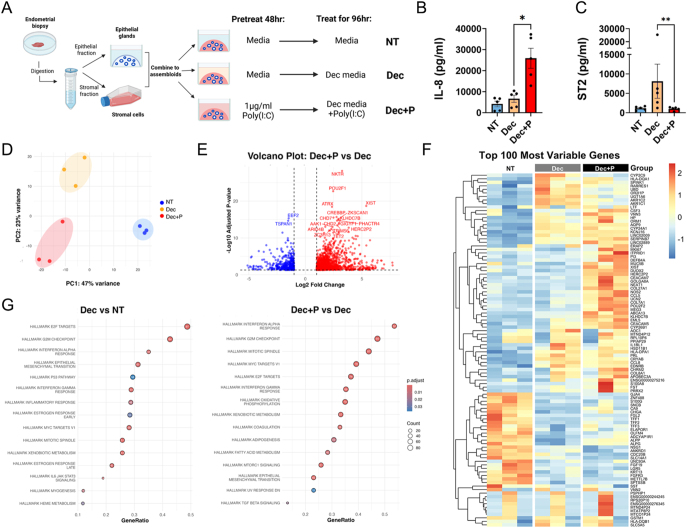
Exposure of patient-derived endometrial assembloids to Poly(I:C) prior to decidualization altered gene expression associated with physiological endometrial functions. (A) Summary of workflow from patient endometrial biopsy to 3D endometrial assembloid culture with treatment timeline. (B and C) ELISA quantification of inflammatory IL-8 and decidualization marker, ST2/IL-1RL1 (pg/mL, *n* = 5). (D) Principal component analysis of transcriptome-wide expression changes between treatments (*n* = 3 patients); samples colored by group: NT (blue), Dec (orange), and Dec + P (red). (E) Volcano plot showing the top 50 gene changes for Dec + P versus Dec treatment. (F) Heatmap of the top 100 most significant genes across NT (white), Dec (gray), and Dec + P (black) treatments. (G) Gene set enrichment analysis using MSigDB of differentially expressed genes between Dec vs NT and Dec + P vs Dec. *P < 0.05.

To characterize the impact of viral dsRNA exposure on endometrial function, we used an endometrial assembloid model that consists of primary human endometrial epithelial cells organized in 3D gland-like organoids, surrounded by patient-matched endometrial stromal cells. These assembloids can undergo decidualization and functionally support the expansion of human blastocysts (Rawlings *et al.* 2021), demonstrating their relevance for studying endometrial decidualization and function *in vitro*. To determine if prior Poly(I:C) exposure affects decidualization, undifferentiated assembloids were first exposed to Poly(I:C) for 48 h to induce a proinflammatory response and then treated with a deciduogenic hormonal cocktail (Dec) for four days (Fig. 2A). Compared to non-exposed control cultures, Poly(I:C) exposure significantly increased IL-8 secretion in decidualized assembloids (Fig. 2B) in parallel with reduced secretion of sST2, a soluble decoy receptor for IL-33 that is typically upregulated during decidualization (Salker *et al.* 2012) (Fig. 2C). RNA sequencing identified 2,984 differentially expressed genes across assembloid treatments (NT, Dec, and Dec + P). Principal component analysis demonstrated clear separation among treatment groups, consistent with transcriptional reprogramming (Fig. 2D and F). The most significantly upregulated genes in the Dec + P group compared to the Dec group were: *NKTR, POUZF1, XIST,* and *ATRX*, while the most significantly downregulated were *EEF2* and *TSPAN1* (Fig. 2E). Gene set enrichment analysis identified inflammation, interferon-α and interferon-γ response, and epithelial–mesenchymal transition pathways to be altered in both Dec vs NT and Dec + P vs Dec comparisons (Fig. 2G).

### Prior exposure to Poly(I:C) increased inflammation and inhibited decidualization *in vivo*

**Figure 3:**
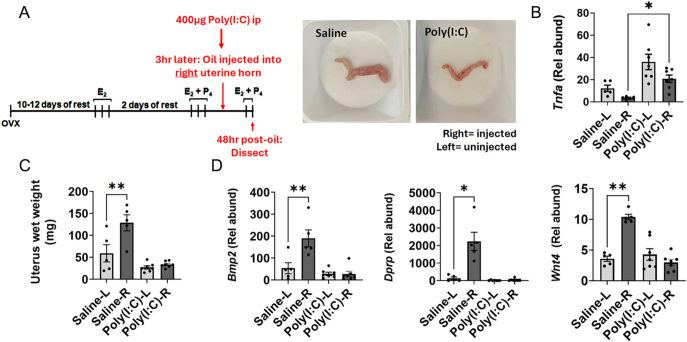
Poly(I:C) induces inflammation and prevents decidualization in a humanized mouse model of decidualization. (A) Schematic summarizing treatment regimen for mouse model and representative photos of uteri at the end of the experiment. (B, C, D) Comparison of changes in gene expression and wet weight of left uninjected (light gray) and right injected (dark gray) horns of saline and Poly(I:C) exposed mice. (B) Uterine *Tnfa* expression. (C) Wet weight of dissected uterine horns. (D) Uterine expression of decidualization markers: *Bmp2, Dprp,* and *Wnt4* (*n* = 5). *P < 0.05, **P < 0.01.

To determine if prior exposure to viral dsRNA can also inhibit decidualization *in vivo*, an established mouse model of decidualization was employed (Fig. 3A) (Whirledge *et al.* 2015, Tong *et al.* 2022). Briefly, 400 μg Poly(I:C) (20 mg/kg) was injected systemically to induce a robust inflammatory response. Then, 3 h later, decidualization was induced by intrauterine oil administration into the right uterine horn. Since decidualization only occurs in the oil-injected horn, the left uninjected horn can act as a matched endogenous control. Systemic Poly(I:C) exposure increased proinflammatory *Tnfa* expression in both the left (undecidualized) and right (decidualized) uterine horns (Fig. 3B). *Tnfa* levels were significantly higher in the decidualized uterine horn of Poly(I:C)-exposed mice compared to that from saline control mice (6.1 ± 1.0-fold, Fig. 3B). Mirroring what we had observed *in vitro*, Poly(I:C) exposure prior to induction of decidualization significantly inhibited decidualization responses in mice (Fig. 3C and D). While in control mice injected with saline, intrauterine oil administration into the right uterine horn led to significant increases in uterine weight and expression of decidualization markers *Bmp2, Dprp,* and *Wnt4* compared to the left uninjected horn, as one would expect during decidualization; these increases were completely abrogated in mice exposed to Poly(I:C) (Fig. 3C and D).

### Preimplantation exposure to Poly(I:C) reduced decidualization, litter size, and fetal growth *in vivo*

**Figure 4:**
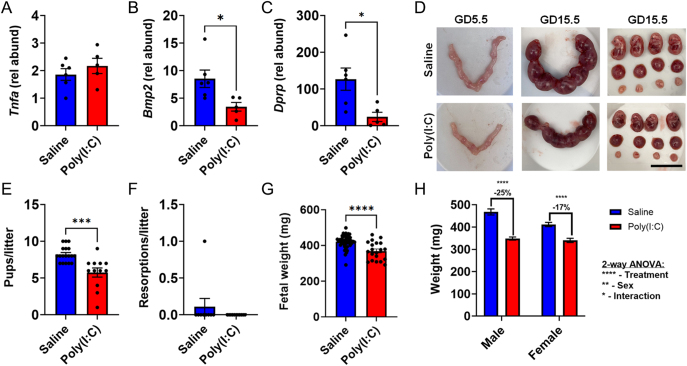
Preimplantation exposure to Poly(I:C) inhibited decidualization and reduced litter size and fetal weight in a sex-specific manner. (A, B, C) Uterine gene expression on gestational day (GD) 5.5 of (A) inflammatory *Tnfa* and decidualization markers, (B) *Bmp2*, and (C) *Dprp*. (D) Representative images of uteri and fetal tissues collected on GD5.5 and GD15.5 from saline and Poly(I:C)-exposed dams. Scale bar = 1 cm. (E) Litter size, (F) number of resorptions, and (G) fetal wet weights on GD15.5. (H) Fetal weights subdivided based on sex and maternal Poly(I:C) exposure (*n* = 11–20 per group). *P < 0.05, ***P < 0.001, ****P < 0.001.

To investigate whether preimplantation viral dsRNA exposure can also affect natural decidualization and subsequent pregnancy success, 400 μg Poly(I:C) (20 mg/kg) was injected systemically in time-mated mice on the day of copulatory plug identification (gestational day (GD) 0.5). In these naturally mated mice, decidualization is initiated on GD4.5 following blastocyst adhesion to the uterine wall. Accordingly, on GD5.5, we observed that Poly(I:C)-exposed mice expressed significantly lower levels of decidualization markers, *Bmp2* (59.6 ± 9.1% decrease) and *Dprp* (80.9 ± 9.9% decrease) compared to control mice (Fig. 4B and C). Interestingly, by this time point, we no longer see increased uterine inflammation as measured by *Tnfa* expression (Fig. 4A). This suggests that the downstream effects on pregnancy observed are not directly due to Poly(I:C)-induced inflammation.

On GD15.5, equivalent to mid-third trimester in humans, we observed that the number of pups per litter is significantly decreased in Poly(I:C)-exposed dams compared to controls (5.8 ± 0.6 vs 8.2 ± 0.3, *P* < 0.001); however, the number of resorptions was not significantly increased (Fig. 4D, E, F). Moreover, fetuses from Poly(I:C)-exposed dams were smaller and weighed significantly less than control mice (12.4 ± 2.7% decrease in wet weight, Fig. 4D and G). When we separated weights based on fetal sex, male fetuses were more growth restricted than female fetuses compared to their respective controls (Fig. 4H).

### Preimplantation exposure to Poly(I:C) altered placental development and reduced placental nutrient transporter expression

**Figure 5:**
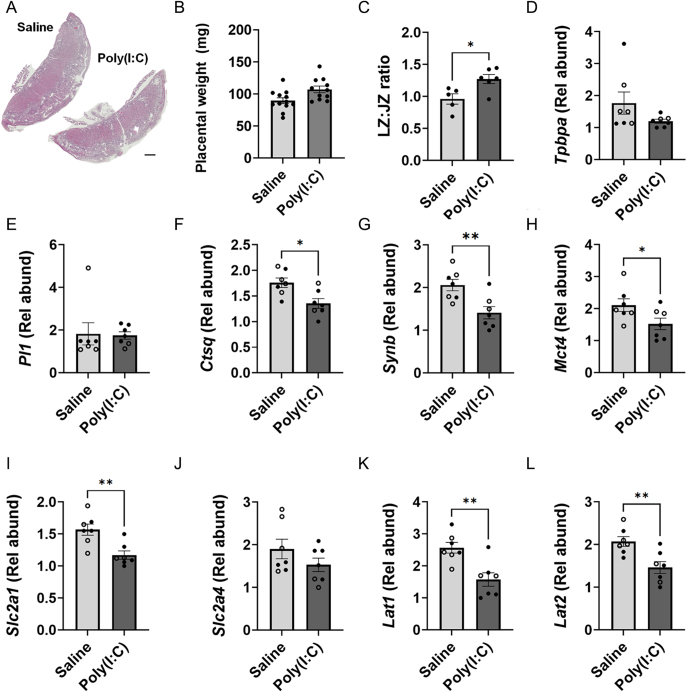
Preimplantation exposure to Poly(I:C) affected subsequent placental development and gene expression at GD15.5. (A) Representative hematoxylin and eosin staining of dissected placentas from saline- or Poly(I:C)-exposed mothers; scale bar = 1 mm. (B) Placental wet weights on GD15.5. (C) Ratio of the measured areas of the labyrinth zone (LZ) and junctional zone (JZ) (*n* = 5). (D, E, F, G, H, I, J, K, L) Gene expression changes in placentas from saline- or Poly(I:C)-exposed mothers using *Tuba* as the housekeeper. Open dots indicate placentas from male fetuses, and solid dots indicate placentas from female fetuses (*n* = 7 placentas from different pregnancies in each treatment group).

The mouse placenta consists of two functional zones: the junctional zone that is closest to the maternal decidua and plays a metabolic and endocrine role; and the labyrinth zone that is primarily responsible for transport between the mother and fetus (Woods *et al.* 2018). While total placental weight at GD15.5 was not significantly different between Poly(I:C)-exposed dams and controls (Fig. 5B), histological analysis revealed that Poly(I:C)-exposed dams had placentas with a reduced junctional zone:labyrinth zone ratio (Fig. 5A and C), indicating altered placental development. Interestingly, placental expression levels of *Tpbpa*, an established marker of the junctional zone, and *Pl1*, a marker of trophoblast giant cells, were not significantly different between saline and Poly(I:C)-exposed dams (Fig. 5D and E). In contrast, preimplantation maternal exposure to Poly(I:C) significantly reduced placental expression levels of *Ctsq*, a marker of sinusoidal giant cells; and *Synb* and *Mct4*, two markers of the syncytiotrophoblast layer II (SynT-II cells, Fig. 5F, G, H).

To further investigate whether preimplantation maternal Poly(I:C) exposure can affect the capacity of the placenta to transport nutrients to the fetus, we quantified placental expression of key glucose and amino acid transporters. We observed that placentas from Poly(I:C)-exposed dams had a 25.3 ± 4.1% decrease in the expression of *Slc2a1* (GLUT1), but no change in *Slc2a4*, encoding GLUT4 (Fig. 5I and J). Preimplantation exposure to Poly(I:C) also reduced expression of L-type amino acid transporters, *Lat1* and *Lat2,* by 38.7 ± 8.5% and 29.4 ± 6.7%, respectively (*P* < 0.05, Fig. 5K and L).

## Discussion

Numerous recent studies have implicated decidual dysfunction in the pathogenesis of obstetrical complications, including preeclampsia and fetal growth restriction (Rabaglino *et al.* 2015, Garrido-Gomez *et al.* 2017, Dunk *et al.* 2019, Yang *et al.* 2023). However, due to ethical considerations and inaccessibility of samples during early pregnancy, whether decidual dysfunction precedes and causes subsequent obstetric disease remains unclear. Using a combination of human endometrial cells cultured in 2D and 3D, as well as mouse models of decidualization and pregnancy, we demonstrate for the first time that preimplantation exposure to viral dsRNA in the mother can significantly impair endometrial decidualization and deleteriously affect subsequent pregnancy outcomes. We observed that maternal inflammation early in pregnancy is associated with later changes in placental development and reduced expression of key glucose and amino acid transporters, leading to growth restriction in pups in a sex-specific manner. While our model cannot rule out the direct impact of maternal inflammation on embryo development, these findings do provide evidence that ripple effects caused by endometrial perturbations prior to implantation can have oversized impacts on the risk of adverse pregnancy outcomes. This provides strong experimental impetus for further research into decidualization and decidual (dys)function in the early pathogenesis of obstetrical complications.

Endometrial stromal cells are the major cell type present in the human and mouse endometrium. When decidualized, these stromal cells are highly secretory and form a central hub for communication with overlying epithelial cells and recruited immune cells to regulate uterine receptivity and function. We demonstrate that pretreatment of human endometrial stromal cells with viral dsRNA significantly induced inflammation and reduced their ability to decidualize in response to estradiol and progesterone. Furthermore, impaired decidualization was also observed in an established assembloid model that consists of endometrial stromal cells cultured in 3D with matched endometrial epithelial glands. This is evidenced by RNA sequencing findings (reduced expression of decidualization genes including *PRL* and *PROK1*) and reduced ST2 secretion. Soluble ST2 is a decoy receptor for the alarmin IL-33 that enables endometrial stromal cells to restrain its initial inflammatory response and its secretion is typically increased during decidualization (Salker *et al.* 2012). We observed that viral dsRNA exposure prior to decidualization reduced ST2 secretion, mirroring what has previously been reported for endometrial stromal cells isolated from patients with recurrent pregnancy loss, compared to healthy controls (Salker *et al.* 2012). While gene set enrichment analysis of differentially expressed genes in the assembloid model was unable to identify changes in pregnancy- or fertility-related pathways, likely due to the small numbers of genes associated with these pathways, we did observe that many of the most significantly upregulated genes in the Dec + P vs Dec group were associated with chromatin remodeling (*ATRX, XIST, CREBBP*), while the most downregulated genes were *EEF2*, which is important for protein synthesis, and *TSPAN1,* which is important for cell–cell interactions.

To further validate the impaired decidualization response observed *in vitro*, we employed mouse models of decidualization and pregnancy. While there are some key differences in mouse decidualization and placentation compared to humans, they have been valuable tools for our understanding of the molecular basis of uterine receptivity, decidualization, and implantation as human tissues are usually inaccessible during this vulnerable time. In mice, physiological decidualization is initiated after physical contact between the embryo and the uterine luminal epithelium on gestational day (GD) 4.5. To study decidualization without the confounding factor of the embryo, we and others have mimicked this contact with an intrauterine oil injection in a humanized mouse model of decidualization (Whirledge *et al.* 2015, Tong *et al.* 2022). This model enables us to study the effects of viral inflammation at any point of the decidualization process, and by injecting oil only into the right uterine horn, the left uterine horn of each mouse can act as an endogenous undecidualized control. Using this *in vivo* model, we showed that early systemic inflammation caused by viral dsRNA robustly inhibits decidualization on both morphological and molecular levels. These observations are also validated in our time-mated mice where physiological decidualization can be monitored by gene expression changes on GD5.5.

Using a novel preimplantation exposure model, we show for the first time that early maternal immune activation and endometrial dysfunction can have subsequent effects on pregnancy outcomes. Since mice have multiple pups per litter, we were able to show that preimplantation maternal viral dsRNA exposure led to reduced implantation rates, as demonstrated by smaller litters but no increase in resorptions in the dsRNA-exposed dams. Clinically, this suggests that early maternal viral inflammation may cause implantation failure rather than miscarriage per se. This observation contrasts with most other inflammatory models of pregnancy where increased resorptions or fetal demise is observed (Gendron *et al.* 1990, Chatterjee *et al.* 2012, Wolfson *et al.* 2015). In those studies, maternal inflammation is induced later in gestation and it is likely that this overt inflammation directly affected embryo development and/or early placental development, leading to increased fetal demise (Reginatto *et al.* 2021, Jeong *et al.* 2023). Indeed, increased uterine secretion of TNF-α in response to bacterial lipopolysaccharide (LPS) on GD12 can cause fetal resorption (Gendron *et al.* 1990). Our model has the unique strength that the induced maternal inflammation is resolved by the time decidualization occurs at GD5.5, as evidenced by a lack of increased uterine *Tnfa* (Fig. 4A). Thus, altered development of the placenta proper from GD8 is likely not confounded by maternal inflammation induced by the initial viral dsRNA exposure, although without embryo transfer studies, we cannot exclude the direct impact of maternal inflammation and viral dsRNA exposure on embryo and trophectoderm development.

In addition to reduced implantation, for conceptuses that did implant in dams previously exposed to viral dsRNA, fetal growth restriction was observed, particularly for male pups. Fetal growth restriction has been similarly reported in other later inflammatory models, for example maternal administration of bacterial LPS in the third trimester (Zhang *et al.* 2022, Jeong *et al.* 2023). In those studies, an increased susceptibility of male fetuses to fetal growth restriction was also noted (Jeong *et al.* 2023), suggesting that male conceptuses and/or their placentas may be more vulnerable than females to maternal inflammation. Clinically, while fetal growth restriction is more common in females (Spinillo *et al.* 1994, Yunis *et al.* 2004), male offspring were reported to be more sensitive to low maternal weight (Spinillo *et al.* 1994). It is also well established that maternal inflammation and stress disproportionately affect male offspring neurodevelopment and behavior compared to female offspring (Bronson & Bale 2014, Han *et al.* 2021). Our current experiments were not sufficiently powered to detect sex-specific differences in placental responses to maternal inflammation; nevertheless, we have retrospectively determined the sex of each analyzed placenta for context and will pursue sex-dependent trophoblast responses to decidual inflammation in future studies.

Both the mouse and human placenta are hemochorial and have trophoblasts directly bathed in maternal blood. The mouse placenta is separated into two functional zones: the junctional zone close to decidua and the labyrinth zone for transport between the mother and fetus. Since we did not see changes in *Tpbpa*, a marker of spongiotrophoblasts and the junctional zone, we focused our attention on the labyrinth zone. In humans, the syncytiotrophoblast is the main cell responsible for transplacental transport and this function is shared by the syncytiotrophoblast I, syncytiotrophoblast II, and sinusoidal trophoblast giant cells in the mouse (Ander *et al.* 2019). In addition, the sinusoidal trophoblast giant cells also carry out some endocrine functions like the human syncytiotrophoblast (Outhwaite *et al.* 2015). Interestingly, while preimplantation maternal inflammation did not affect the overall size of the placenta, early maternal inflammation did lead to reduced expression of markers associated with the syncytiotrophoblast II layer (*Synb* and *Mct4*) and sinusoidal trophoblast giant cells (*Ctsq*). In contrast, *Pl1*, a marker for parietal trophoblast giant cells (equivalent to human extravillous trophoblasts), was not significantly different in viral dsRNA-exposed pregnancies compared to controls, supporting that the differences we observed are unique to specific cell types and functions of the placenta.

In both the human and mouse placenta, GLUT1/*SLC2A1* is the primary, constitutively expressed glucose transporter responsible for the bulk of glucose transport from the mother to her fetus. In comparison, GLUT4/*SLC2A4* expression is regulated by insulin and is responsive to maternal metabolic states, thus playing a secondary role in placental glucose transport (Illsley & Baumann 2020). We observed a significant decrease in *Slc2a1*, but not *Slc2a4*, expression, suggesting that preimplantation maternal inflammation can directly affect the major, steady-state glucose transporter of the placenta. Interestingly, levels of placental GLUT1 expression have been reported to be decreased in preeclamptic placentas (Luscher *et al.* 2017); however, levels and activities are controversial in intrauterine growth restriction (Illsley & Baumann 2020). In our model, *Lat1* and *Lat2*, two L-type amino acid transporters responsible for the transport of essential amino acids, are also significantly reduced with maternal viral dsRNA exposure. Since glucose and amino acids are the main building blocks and energy source for fetal growth *in utero*, we posit that reduced expression of these nutrient transporters led to the fetal growth restricted phenotype observed. In the future, it would be imperative to carry out embryo transfer studies and placental glucose and amino acid transport studies to demonstrate that preimplantation inflammation and altered decidualization in the mother can directly affect subsequently placental transport *in vivo*.

In summary, this study provides the first evidence that prior maternal inflammation can affect decidualization and endometrial function with deleterious effects on subsequent placental development and pregnancy outcomes. Indeed, it has been reported that maternal inflammation in response to HIV is more predictive of fetal growth restriction than placental viral load (Iqbal *et al.* 2010). We also know that miscarriage, potentially indicative of endometrial dysfunction, compounds the risk of obstetrical complications in future pregnancies including fetal growth restriction and preterm birth (Quenby *et al.* 2021). Combined with our study, these lines of evidence provide compelling support for future investigations into the molecular mechanisms by which altered endometrial decidualization and function influence subsequent placental development, including the role of endometrial-derived soluble factors and extracellular vesicles. Advancing our understanding of endometrial dysfunction in the context of obstetric disease pathogenesis will highlight a critical preconception window in women that holds immense diagnostic and therapeutic opportunity and open new avenues for the development of early screening and personalized therapeutic strategies to improve pregnancy outcomes in the future.

## Declaration of interest

The authors declare that there is no conflict of interest that could be perceived as prejudicing the impartiality of the work reported.

## Funding

This work was supported by a K99/R00 Transition to Independence Grant (K99HD101653) and a project grant (R01HD117930) awarded to MT from the Eunice Shriver National Institute of Child Health and Development/National Institutes of Health (NIH), as well as a Wellcome Trust Investigator Award to JJB (212233/Z/18/Z). MT was also supported by a CTSA Grant (UL1TR001863) from the National Center for Advancing Translational Science, NIH, and she is the recipient of the Preeclampsia Foundation Vision Grant 2024 and the Paul Titus Fellowship 2025. TMR was supported by a fellowship from Warwick-Wellcome Trust Translational Partnership initiative. LJ was funded by a Rudolph J Anderson postdoctoral fellowship from Yale School of Medicine. EYH was funded by the Discovery to Cure Summer Internship Program.

## Author contribution statement

JNS, TMR, JJB, ESL, and MT conceived and designed the study. JNS, TMR, LJ, MT, ZT, RG, EYH, PJB, ESL, and MT performed experiments and analyzed the data. JNS, LJ, RG, and MT drafted the manuscript. All authors read and approved the final manuscript.
